# Yellow Fever a Nautical Disease

**Published:** 1879-12

**Authors:** 


### BIBLIOGRAPHICAL.

Yellow Fever a Nautical Disease.—Its Origin and Prevention, by John Gamgee. D. Appleton & Co., New York. 1879.

This volume is written upon the exclusive basis that yellow fever is the product solely of foul ships. As "no recondite research is needed to reveal a new elixir of life," so, in these days, it seems, no great effort is required in the production of various and varied theories, new and startling relative to the origin, cause, treatment and *thorough prevention* of yellow fever. The mass is necessary, however, to present the opportunity for selection, and the time may be when the work before us will be the index to the proper and correct comprehension of this dire epidemic.

